# When Should Clinical Mycology Laboratories Perform Antifungal Susceptibility Testing? Revisiting Practice Through the Lens of Intrinsic Resistance

**DOI:** 10.1007/s11046-025-01033-6

**Published:** 2025-12-21

**Authors:** Cornelia Lass-Flörl, Sevtap Arikan-Akdagli

**Affiliations:** 1 Institute of Hygiene and Medical Microbiology, ECMM Excellence Center of Medical Mycology, Medical University of Innsbruck, Innsbruck, Austria; 2 Faculty of Medicine, Department of Medical Microbiology, Hacettepe University, Ankara, Türkiye

**Keywords:** Intrinsic resistance, Acquired resistance, Natural resistance, Resistance, Fungi

## Abstract

**Background:**

Antifungal susceptibility testing (AFST) guides therapy for invasive and refractory fungal infections, but routine testing of species with predictable, species-level (intrinsic) resistance can waste laboratory resources.

**Methods:**

Narrative synthesis of guideline documents and recent literature to define intrinsic resistance, summarize major fungal examples, and propose a selective AFST framework.

**Results:**

Key taxa show intrinsic non-susceptibility (e.g., Pichia kudriavzevii to fluconazole; Mucorales to short-tailed azoles; Cryptococcus spp. to echinocandins). Terminology differences between EUCAST and CLSI are noted. Recommended AFST is targeted to invasive/rare pathogens, treatment failure, surveillance, outbreak investigation, and evaluation of novel agents.

**Conclusion:**

A selective AFST strategy, reserving routine testing for species with variable or emergent resistance, enhances laboratory efficiency and antifungal stewardship while ensuring appropriate clinical therapy.

## Introduction

Antifungal susceptibility testing (AFST) is a critical tool in managing invasive and refractory fungal infections, especially in high-risk patients or when therapeutic failures occur [1, 2]. The current European Committee on Antimicrobial Susceptibility Testing (EUCAST) and the Clinical and Laboratory Standards Institute (CLSI) guidelines recommend AFST for invasive infections, uncommon pathogens, surveillance, outbreak investigations, and suspected acquired resistance [3–5]. However, routine AFST for fungi with intrinsic resistance often offers little clinical benefit but laboratory resources [6]. Hence, here we give a breakdown of the right approach on fungi that should not be routinely tested with a main focus on intrinsic resistance. However, this framework presupposes access to rapid, accurate species-level fungal identification which is still not the reality in many regions. In numerous routine clinical laboratories, mycology expertise and diagnostic infrastructure remain limited, so any move towards selective AFST must be coupled with efforts to strengthen fungal identification or ensure access to reference centres.

Intrinsic resistance is widespread, affecting numerous antifungal agents and fungal taxa [7]. Even recently introduced therapies such as olorofim and fosmanogepix exhibit predictable intrinsic‑resistance spectra. *Cryptococcus* spp., Mucorales (e.g., *Rhizopus* and *Mucor* spp.), *Trichophyton* spp., and *Pneumocystis jirovecii* show natural resistance to olorofim because of differences in the formate dehydrogenase 1 enzyme or alternative metabolic pathways [6]. Similarly, fungi that lack the Gwt1‑dependent GPI‑anchor biosynthesis pathway—or possess alternative mechanisms for cell‑wall integrity—are intrinsically resistant to fosmanogepix; this includes Mucorales, *Cryptococcus, Fusarium*, and *Pneumocystis* spp. [8, 9].

This commentary outlines a selective AFST framework focusing on fungi with intrinsic resistance to specific antifungal classes. We consistently use the term intrinsic resistance—preferred in clinical practice—to describe species- or genus-level non-susceptibility encoded in the fungal genome and present in all isolates, irrespective of prior exposure. Acquired resistance, by contrast, arises via mutation or adaptive mechanisms following antifungal exposure [1].

### Defining Resistance Types

*Intrinsic resistance.* A species- or genus-level trait whereby most or all strains are non-susceptible to a drug class due to inherent genetic or structural features, independent of drug contact [2, 6]. In the past, the terms “primary”, “innate” or “inherent” resistance were used interchangeable. CLSI continues to use the formal label “Intrinsic resistance” for natural, species-wide antifungal non-susceptibility [10]. The definition explicitly equates intrinsic, inherent and innate resistance. This definition highlights that intrinsic resistance reflects the normal (wild-type) MIC distribution for the species, such that susceptibility testing is usually unnecessary when a fungus is known to be intrinsically resistant to a drug.

EUCAST has taken a different terminological approach in recent years and decided to abandon the term “intrinsic resistance” in its official communications [11]. EUCAST introduced the concepts of “expected resistant phenotype” and “expected susceptible phenotype” across all subcommittees. In this schema, a species is assigned an expected resistant phenotype for a particular drug if the great majority of wild-type isolates (e.g. ≥ 90%) are resistant at clinically relevant exposures. These terms allow laboratories to infer resistance or susceptibility based on species identification alone, without performing a test, because the outcome is expected given the species’ known biology. For example, rather than saying *Pichia kudriavzevii* (*Candida krusei*) has “intrinsic resistance” to fluconazole, EUCAST would classify *P. kudriavzevii* as having an expected resistant phenotype to fluconazole, meaning one can assume it will test resistant. It can be expected that the EUCAST documents on fungi will be gradually aligned with the new terminology.

*Acquired resistance.* Resistance emerging within a previously susceptible species following drug exposure and selective pressure [1, 12].

### Key Features of Intrinsic Resistance in Medically Important Fungi

Species- or genus-specific. All members of a species (e.g., *P. kudriavzevii* to fluconazole) or entire genera (e.g., most *Aspergillus* spp. to fluconazole) exhibit non-susceptibility [1, 2, 6].

Genetic basis. Mechanisms—target site alterations, efflux pump expression, or target modifications—are genomically encoded and vertically inherited [9, 13].

Independence from drug exposure. Intrinsic resistance exists without prior antifungal therapy [6].

### Examples of Intrinsic Resistance and the Underlying Mechanisms

#### P. kudriavzevii

Erg11 (Cyp51p) enzyme shows multiple amino-acid differences from that of *Candida albicans* and other susceptible species, markedly reducing fluconazole binding affinity while still processing lanosterol to ergosterol [6, 13]. Overexpression of ABC/MFS pumps and rerouting of sterol intermediates may further diminish drug impact.

#### Aspergillus terreus

Elevated amphotericin B MICs are tied to membrane composition and stress-response differences [14–16]. Unlike other Aspergilli, *A. terreus* constitutively expresses high levels of catalases and Hsp90-dependent stress-response pathways that mitigate amphotericin B-induced oxidative damage, tipping the balance away from lethal reactive-oxygen-species accumulation.

Common examples for cryptic *Aspergillus* species for which high MICs are obtained suggesting intrinsic resistance are *Aspergillus alliaceus* (in Section Flavi) for amphotericin B, *Aspergillus lentulus* (in Section Fumigati) and possibly *Aspergillus tubingensis* (in Section Nigri) for azoles [17]. Amphotericin-B failure in *A. alliaceus* appears to stem from an atypical sterol envelope that binds the polyene poorly [17]; azole resistance in *A. lentulus* is driven mainly by a naturally low-affinity Cyp51A, whereas in *A. tubingensis* it is point mutations—especially H467Q—in Cyp51A (plus minor Cyp51B input) that blunt triazole activity [17]. *A. tubingensis* repeatedly shows species-wide elevated itraconazole/voriconazole MICs but more data are needed for official classification as “intrinsically azole-resistant”.

#### Mucorales (e.g. *Rhizopus *spp., *Mucor* spp.)

These species harbor two CYP51 paralogs (CYP51 F1 and F5) with a conserved amino-acid substitution (e.g., Y129F in *R. arrhizus/R. oryzae*) within the enzyme’s active-site. “BC loop” sterically hinders binding of short-tail azoles (fluconazole, voriconazole) while preserving catalytic activity [18, 19]. Some CYP51 paralogs preferentially demethylate eburicol or obtusifoliol rather than lanosterol, further reducing azole efficacy against their primary substrate.

*Rasamsonia argillacea* complex species*,* previously named as *Geosmithia* spp*.* Taxonomic reclassification revealed unique Cyp51A variants with multiple substitutions in the heme-binding pocket, resulting in very high voriconazole and isavuconazole MICs (> 32 mg/L) and cross-resistance among short- and long-tail triazoles [20].

#### *Cryptococcus* spp.

Intrinsic echinocandin resistance is due to impaired drug access and altered membrane dynamics. For example, a mutation in the lipid‐flippase subunit Cdc50 blocks caspofungin uptake and additional stress-response and cell-wall remodeling pathways further reduce echinocandin activity in *C. neoformans* [21].

### Gradations of Susceptibility

Some species display intrinsic reduced susceptibility rather than intrinsic resistance (e.g., *Nakaseomyces glabrata* (*Candida glabrata*)—susceptible/increased exposure category for fluconazole). [22]. There is no susceptible category for fluconazole and *N. glabrata*. Most *N. glabrata* isolates fall into the susceptible, increased exposure category and the remaining are resistant to fluconazole. The susceptible, increased exposure category for fluconazole reflects the need for dosing adjustments in treatment.

*Candida parapsilosis* on the other hand, shows reduced echinocandin susceptibility due to FKS1 polymorphisms. There are susceptible and resistant categories for echinocandins against *C. parapsilosis* and clinically it may still respond to standard echinocandin therapy despite the reduced susceptibility, underscoring the value of AFST in these contexts [7].

### Selective AFST Recommendations for the Medical Mycology Routine Laboratory

Routine AFST indicated. All invasive infections, rare or emerging pathogens, surveillance, outbreak settings, lack of response to treatment, and whenever acquired resistance is suspected [1–3]. Recent global and regional studies have documented an increasing burden of superficial dermatophyte infections accompanied by the emergence of antifungal resistance, most prominently terbinafine-resistant *Trichophyton indotineae* within the *Trichophyton mentagrophytes/interdigitale* complex. [24]. Terbinafine resistant *Trichophyton rubrum* strains, on the other hand, have also been reported at varying rates [25, 26]. These data underline that AFST for dermatophytes, using standardized EUCAST or CLSI methodologies, is increasingly relevant at least for refractory or widespread superficial infections and for surveillance in regions where resistant strains are emerging. Recently, EUCAST- based agar-screening method was also developed and evaluated in a multicenter study, validating its reliable utility for screening terbinafine and itraconazole susceptibility of *Trichophyton* spp. [27].

Routine AFST unnecessary. Species with well-established intrinsic resistance patterns (e.g., *P. kudriavzevii* to fluconazole; Mucorales to short-tailed azoles), unless atypical susceptibility patterns arise.

Resource optimization. Laboratories can reallocate efforts toward pathogens with variable or emerging resistance profiles. An overview of fungal pathogens with documented intrinsic resistance against important antifungal drugs is given in Table 1. Implementation of a selective AFST strategy should, however, be context-dependent: in well-resourced centres with reliable, rapid species-level identification (e.g. MALDI-TOF MS and, where needed, sequencing), algorithms that omit testing of intrinsically resistant species are reasonable, whereas in laboratories with limited identification capacity, broader AFST or referral of isolates to reference laboratories may be necessary until robust fungal identification can be ensured.

**Table 1:** Overview of documented intrinsic resistance against a wide range of fungal pathogens (data compiled from [8, 9, 15, 20])

**Fungal pathogens**	Amphotericin B	Fluconazole	Voriconazole	Isavuconazole	Posaconazole	Itraconazole	Opelconazole	Anidulafungin	Caspofungin	Micafungin	Rezafungin	Olorofim	Fosmanogepix	Ibrexafungerp	Flucytosine
*Candida* species												**X**			
***Pichia kudriavzevii***		**X**										**X**	**X**		**X**
*Cryptococcus gattii & deuterogattii*								**X**	**X**	**X**	**X**	**X**			
*Cryptococcus neoformans*								**X**	**X**	**X**	**X**	**X**			
*Rhodotorula* species								**X**	**X**	**X**	**X**	**X**			
*Trichosporon* species		**X**						**X**	**X**	**X**	**X**	**X**			
*Aspergillus species*		**X**													
*A. terreus* species complex	**X**	**X**													**X**
*A. alliaceus*	**X**	**X**													**X**
Mucorales		**X**	**X**				**X**	**X**	**X**	**X**	**X**	**X**		**X**	**X**
*Fusarium* species		**X**						**X**	**X**	**X**				**X**	**X**
*Scedosporium* species	**X**	**X**												**X**	**X**
*Lomentospora prolificans*	**X**	**X**						**X**	**X**	**X**				**X**	**X**
*Purpureocillium lilacinum*	**X**	**X**												**X**	**X**
*Scopulariopsis* species		**X**													***X***
*Rasamsonia argillaceae* species complex		**X**	**X**	**X**											
*Malassezia furfur*												**X**			**X**

The marking (X) implies intrinsic resistance. For the remaining (unmarked) drug-bug combinations, the susceptibility profile has either been explored and no intrinsic resistance has been documented or no data have yet been reported

### Exceptions Warranting AFST in Intrinsically Resistant Fungi

Unanticipated acquired resistance to another antifungal drug in an intrinsically resistant species (e.g., echinocandin-resistant *N. glabrata*) [1, 3].

Confirmation of intrinsic resistance in clinical setting or the initial evaluations of the degree and spectrum of in vitro activities for novel antifungal agents [5].

Periodic surveillance for any possibly existing intrinsic resistance as new drugs are developed and introduced to clinical practice (e.g., olorofim, fosmanogepix) [12, 23].

### Clinical Implications and Guidelines

Clinical practice guidelines incorporate intrinsic resistance by advising “against” the use of the agent for which intrinsic resistance is observed and recommending alternative antifungal drugs that are known to be effective for the species in question (e.g., “against” for fluconazole and recommendation of echinocandins or amphotericin B for *P. kudriavzevii*) [2]. Vigilance for intrinsic resistance to emerging therapies will optimize salvage regimens and guide new-agent development. Awareness of intrinsic resistance to novel agents (e.g., olorofim, fosmanogepix) is crucial as these drugs enter salvage therapy regimens. In Table 1 we list also rare representatives as well as novel drugs. In addition, the growing recognition of dermatophyte resistance, particularly terbinafine-resistant *T. indotineae* and related genotypes, illustrates that the principles discussed for invasive mycoses also extend to superficial infections: AFST is not required for every case of tinea or onychomycosis, but becomes important in recalcitrant disease, outbreaks, or when first-line therapy fails [24–27].

## Conclusion

A strategy of selective AFST—reserving routine testing for species with variable susceptibility or potential acquired resistance—enhances laboratory efficiency and supports antifungal stewardship. Clear codification of intrinsic resistance ensures clinicians select effective therapies while conserving resources. Importantly, such a strategy is only safe when implemented alongside accurate and timely fungal identification; where mycology infrastructure is limited, selective AFST algorithms must be adapted and supported by reference laboratories. In summary, this review brings together current concepts of intrinsic resistance, practical examples across major fungal taxa, and context-dependent recommendations for when AFST should and should not be performed, with the aim of helping clinical mycology laboratories prioritize testing where it adds the greatest value for patient care and antifungal stewardship.

## Data Availability

No datasets were generated or analysed during the current study.
